# Seroprevalence of SARS-CoV-2 Infection among Occupational Groups from the Bucaramanga Metropolitan Area, Colombia

**DOI:** 10.3390/ijerph18084172

**Published:** 2021-04-15

**Authors:** Claudia C. Colmenares-Mejía, Norma Serrano-Díaz, Doris C. Quintero-Lesmes, Ligia Meneses, Isail Salazar Acosta, Álvaro J. Idrovo, Duván Y. Sanabria-Echeverry, Helmer Cordero-Rebolledo, Víctor Castillo

**Affiliations:** 1 Research Centre, Fundación Cardiovascular de Colombia, Floridablanca 681004, Colombia; normaserrano@fcv.org (N.S.-D.); dorisquintero@fcv.org (D.C.Q.-L.); isailsalazar@fcv.org (I.S.A.); 2 Clinical Laboratory, Fundación Cardiovascular de Colombia, Floridablanca 681004, Colombia; ligiameneses@fcv.org; 3 Public Health Department, Universidad Industrial de Santander, Bucaramanga 680006, Colombia; idrovoaj@uis.edu.co; 4 School of Civil Engineering, Universidad Industrial de Santander, Bucaramanga 680006, Colombia; duvansan@uis.edu.co (D.Y.S.-E.); helmercordero@gmail.com (H.C.-R.); 5 CEO, Fundación Cardiovascular de Colombia, Floridablanca 681004, Colombia; victorcastillo@fcv.org

**Keywords:** seroepidemiologic studies, prevalence, coronavirus infections, occupational exposure, occupational health

## Abstract

The negative effects of coronavirus disease 2019 (COVID-19) pandemic have impacted the world economy due to the absence from work because of SARS-CoV-2 infection in workers, among other reasons. However, some economic areas are essential to society and people must continue working outside the home to support economic reactivation; their serological profile could be different from that of the global population. Cross-sectional study: Workers from health, construction, public transportation, public force, bike delivery messengers, independent or informal commerce areas, and residents of Bucaramanga or its metropolitan area were invited to participate. All participants self-completed a virtual survey and a blood test was taken to assess IgG and IgM with the ARC COV2 test. Seroprevalence was estimated considering a complex survey design, correcting for a finite population effect and adjusting for test performance. A total of 7045 workers were enrolled; 59.9% were women and most were residents of Bucaramanga and working in health occupations. The global adjusted seroprevalence was 19.5% (CI: 95% 18.6–20.4), being higher for Girón (27.9%; 95% CI: 24.5–31.30). Workers with multiple contact with people during working hours or using public transportation to go to work had a higher frequency of seropositivity for SARS-CoV-2. The seroprevalence among workers living in these four municipalities from the Colombian northeast area is still low.

## 1. Introduction

The coronavirus disease 2019 (COVID-19) pandemic has direct effects not just in terms of infection, illness, and death in many individuals around the world, but also on the economy; since the early implementation of governmental measures of obligatory social isolation to flatten the curve, the decrease in work activities has generated serious economic shrinking with respect to the pre-pandemic state [1] in several countries. Some sectors have been close to collapse (car sales, 92%; restaurants sales, ~95%) [2], including those in Latin America and the Caribbean. [3] For this region, the World Bank projected a fall of −7.2% (with a growth of 2.8% in 2021) and *Comisión Económica para América Latina y el Caribe* (CEPAL) estimated a contraction of −9.1% in 2020 [4,5].

For Colombia, the World Bank projected a fall of −4.9% and CEPAL forecasted a contraction of −5.6% of Colombian economy in 2020 [5]; moreover, the governmental measures affected 9.2 million workers directly and the National Republic Bank estimated economic losses between 4.6 and 59 billion pesos per month [6], where services (accommodation and food, real estate, administrative services, professional and technical activities, construction, and commerce) were the most affected economic area. However, a group of workers had to be active throughout the pandemic because they are essential to society. Working outside the home amid the COVID-19 pandemic is an unavoidable activity for some occupational groups who cannot work from their homes. Moreover, in developing countries, this may be more necessary due to the high occurrence of informal work with precarious labor conditions. The informal economy includes workers employed by formal, registered firms on a casual, daily wage basis, as well as subsistence actors such as self-employed workers and workers involved in informal enterprises [7].

Even though knowing the proportion of people infected by SARS-CoV-2 allows us to know the real burden of COVID-19, as well as to identify the exposure factors associated with infection to support public health decision-making [8,9], studies have focused mainly on high-risk populations [10,11] or on the general population [12], finding important differences in prevalence estimates [12]. This is probably due to the location, moment of the epidemic curve, type of population, and antibody evaluation test, among others. Furthermore, we believe that the differences in exposure probability could be related to occupational groups or professions.

**Figure 1:**
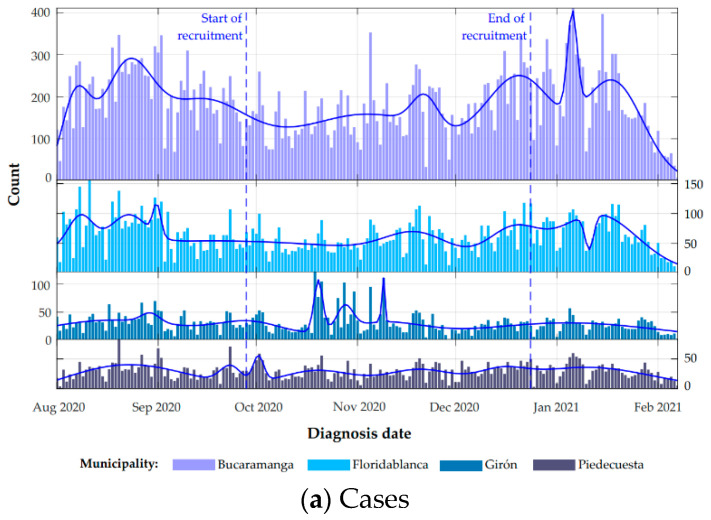

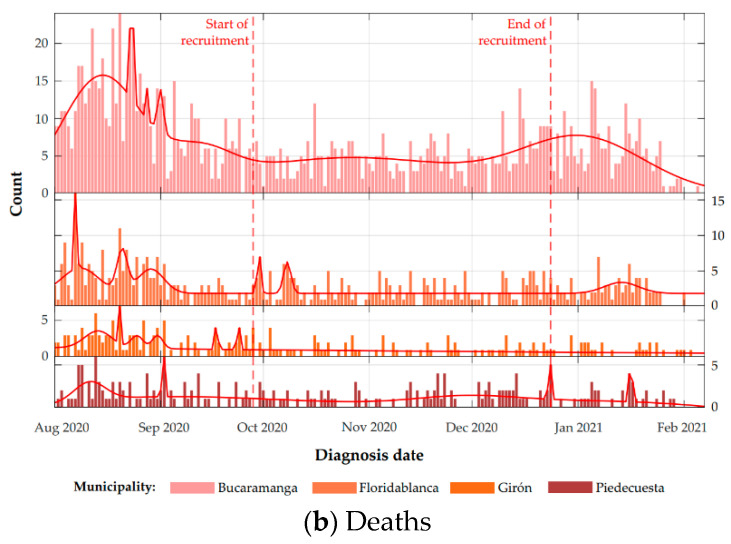
Reported SARS-CoV-2 daily confirmed cases and deaths for the municipalities of the Bucaramanga metropolitan area. (**a**) New daily confirmed cases, (**b**) Daily deaths. Dashed lines delimit the frame time in which recruitment was carried out (28 September and 24 December of 2020). Data source: Colombia National Health Institute; https://www.ins.gov.co/Noticias/Paginas/coronavirus-casos.aspx (accessed on 20 March 2021).

In Colombia, the government decreed a nationwide lockdown in March 2020 and was allowing the economic reactivation of various companies, in accordance with the capacity to fulfill biosafety protocols. This restart had important milestones on 1 June and 1 September 2020 due to the large number of permitted economic activities. In the case of Santander, a department located in the northeast of the country, until June, it was evident that the population strictly complied with the confinement [13]. Then, there was a very rapid increase in the number of cases until it reached its peak in August 2020 (Figure 1a) that was not detected by the public health surveillance system. These differences from the rest of the country, despite the low number of tests analyzed and the evidence suggesting low quality of public health surveillance data [14], raised many doubts about the real situation of the pandemic in the Bucaramanga metropolitan area. The seroprevalence in Bucaramanga metropolitan area is unknown, especially among the workers who have worked during the pandemic. Therefore, the aim of this study was to estimate the seroprevalence of SARS-CoV-2 infection among workers from different occupational groups in the Bucaramanga metropolitan area (Santander, Colombia) who have been active outside the home throughout the pandemic.

## 2. Materials and Methods

### 2.1. Study Design and Population Recruited

An observational cross-sectional study with prospective data collection was developed in the Bucaramanga metropolitan area (Santander, Colombia), consisting of three other municipalities (Floridablanca, Girón, and Piedecuesta). This region is inhabited by 1,111,999 people according to the National Population and Housing Census carried out in 2018 [15]. Adults (>18 years old) who were residents of Bucaramanga or its metropolitan area since August 2020 and who were formally employed in any of the following occupational groups were invited to participate: Health, construction, public transportation (bus and taxi drivers), public forces (army, police, and transit officers), bike delivery messengers, independent workers, or part of informal commerce (including shopkeepers in grocery stores).

### 2.2. Sampling Methods

For formal employment, a stratified sampling by occupational groups according to the census from the Bucaramanga Commerce Chamber was carried out. All legally constituted companies within these groups were identified and those selected were invited to participate; if they accepted, the study information was sent and disclosed among employees for voluntary participation. For informal employment, convenience sampling was carried out in public markets, grocery stores, and neighborhoods with a higher proportion of confirmed COVID-19 cases. Recruitment was carried out between 28 September and 24 December of 2020. A total of 7000 participants were recruited, which allowed to estimate a seroprevalence of 20% with a precision of 0.9% and a 95% confidence level.

### 2.3. Data Collection and Variables Measured

All participants self-completed an online survey that included sociodemographic data (age, marital status, education level, socioeconomic strata, and address), occupational sector (health, public transportation, public force, public services, security, construction, food, education, grocery store tenants/informal commerce, independent worker, administrative/municipal services, cleaning, bike deliveries workers, or another), cigarette smoking status, medical history (presence of stroke, hypertension, acute myocardial infarction, dyslipidemias, diabetes mellitus, chronic obstructive pulmonary disease (COPD/asthma), obesity, non-skin cancer, HIV/AIDS, and autoimmune diseases), possible contact with people with suspected or confirmed COVID-19 infection, presence of symptoms since March 2020 (cough, fever >38 °C, chills, fatigue, myalgia, shortness of breath, wheezing, chest pain, headache, odynophagia, dizziness, rhinorrhea, diarrhea, nausea or vomiting, hemoptysis, nasal congestion, and anosmia), information about possible exposure to infection, such as type of transportation used to go to work or to assist in medical consultations, use of personal protective equipment (gloves, conventional mask, N95 mask, specific clothes or shoes to go out on the street, glasses, face mask, and hat or hair up), prevention activities (bathing when entering the home, washing hands upon arrival at destination, washing hands every two hours, using antiseptic gel, and keeping a distance of at least two meters from other people), quarantine (if symptoms or a positive polymerase chain reaction (PCR) test), rapid tests previously performed, COVID-19 infection confirmed by PCR after the beginning of symptoms, and hospitalization or intensive care unit (ICU) stay. The study data were collected and managed using REDCap electronic data capture tools hosted at Fundación Cardiovascular [16,17]. Electronic informed consent was obtained from all of the subjects involved in the study. This consent was available to be downloaded and saved by each participant.

### 2.4. Geolocation

Participants’ addresses were collected in a parameterized way (type, number, suffix and cardinal direction of the main and secondary roads, license plate number, neighborhood, city, department, and country). Then, the address was standardized with a road or intersection type for each participant according to the world composite geocoder in ArcGIS online. The ArcGIS world geocoding service for Colombia offers level 2 or good quality, which refers to the degree of street-level address coverage in the country [18]. First, ArcGIS tools were used to convert the REDCap database to an ArcGIS geodatabase file; second, each attribute of the address, neighborhood, city, department, and country data were matched against the fields of ArcGIS world composite geocoder; third, the batch geocoder was executed, which consisted in transforming the address data into point-like geographic coordinates on the map for each record of the data batch, establishing the real position of each participant on the geographic territory. Finally, a spatial database was generated, where each record had a score between 0 and 100 (100 being the best accuracy) that indicated the degree of agreement with the address. For records with scores <100, a manual geolocation debugging was performed, verifying the location on the base cartography maps in ArcGIS online and geographic information systems (Supplemental Figure S1 shows the flow of each of the stages for geolocation).

### 2.5. IgG and IgM Measurement

A peripheral blood sample (5 mL) was obtained by venipuncture in the forearm for every participant. The sample was transported from the sampling site to the clinical laboratory of the Fundación Cardiovascular de Colombia to perform Immunoglobulin G (IgG) detection by chemiluminescence assay and Immunoglobulin M (IgM) by enzymatic fluorescence immunoassay. The ARC COV2 test from Abbot^®^ was used for immunoglobulin assessment. This test reports a qualitative result (positive/negative for each antibody). Positive results, either IgG only, IgM only, or both, were reported to SISMUESTRAS (https://apps.ins.gov.co/sismuestras, accessed on 23 March 2021) from *Instituto Nacional de Salud* (INS) as a complementary measure for possible case identification, given the high underestimation found in Bucaramanga metropolitan area and Colombia [19]. Participants that were only IgM-positive were immediately informed through the email address recorded in the virtual survey and were also reported to the Health and Safety at Work Department of their companies (for formally employed workers) to assess the need to confirm a possible infection with PCR.

### 2.6. Statistical Methods

Variables are reported as means with 95% CIs and absolute and relative frequencies. Prevalence was estimated as the number of positive participants (either for IgG, IgM, or both) for the numerator to the total number of participants as the denominator. Additionally, seroprevalence is also presented according to positive only for IgG, positive for IgG and IgM, and positive only for IgM (Table 1 and Table 2). The dataset was declared as a survey (*svyset*), probability weights (*pweights*) were estimated by municipality as *N*/*n*, where *N* = the number of people between 18 and 85 years old in the population and *n* = the number of participants in our sample, and strata were defined according to occupational sector. Finite population correction (FPC) was also estimated using the ((*N* − *n*)/(*N* − 1))1/2 formula, where *N* = the number of people between 18 and 85 years old in the population and *n* = the number of participants in the sample. The primary sampling units (PSUs) were municipalities (Bucaramanga, Floridablanca, Girón, and Piedecuesta). Additionally, prevalence was adjusted by test performance (sensitivity, 85.2%; specificity, 97.3%) [20] using the formula proposed by Sempos and Tian as adjusted prevalence = crude prevalence + specificity − 1/sensitivity + specificity − 1 [21]. The effect of recruitment day (independent variable) on the test results (outcome variable) was assessed through Poisson regression (*svy: poisson*) adjusting for municipalities, gender, occupational sector, and age. Statistical analysis was conducted in Stata 15 [22].

Institutional review board statement: This study was conducted according to the guidelines of the Declaration of Helsinki, and was approved by the ethics committee of Fundación Cardiovascular de Colombia (protocol code CEI-2020-01485, 17 September 2020).

## 3. Results

A total of 7045 workers were included in this study, with a greater proportion of women, residents of Bucaramanga, and health workers (Table 1). During the recruitment period, there was no evidence of a peak in the report of new confirmed SARS-CoV-2 cases or deaths in the four municipalities (Figure 1a,b).

### 3.1. Spatial Characterization

**Figure 2:**
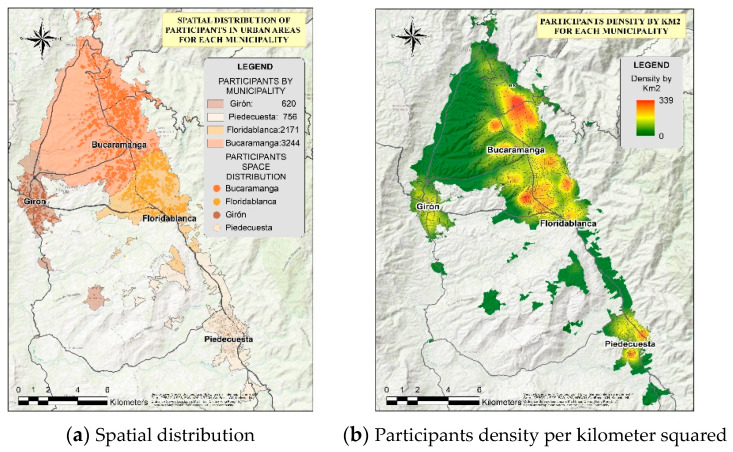
Participants’ geolocation in the Bucaramanga metropolitan area. (**a**) Spatial distribution of participants and density per kilometer squared in urban areas for the municipalities of the Bucaramanga metropolitan area. (**b**) Participant density per kilometer squared for each municipality.

The highest participation was concentrated in Bucaramanga, followed by Floridablanca, Piedecuesta, and Girón (Figure 2a). Figure 2b shows the density map that identifies city areas with the highest number of participants located in urban area per kilometer squared in each municipality; a higher concentration of participation is shown in Bucaramanga, specifically toward the east of the municipality, while in Girón, participants were more dispersed.

### 3.2. Clinical Data

Most participants did not report active cigarette consumption and less than 20% had previous medical conditions (Table 2); the most frequent chronic disease was hypertension (10.0%), followed by COPD/asthma (9.6%), dyslipidemia (6.0%), obesity (5.9%), autoimmune diseases (4.6%), diabetes mellitus (2.5%), non-skin cancer (1.9%), acute myocardial infarction (0.5%), stroke (0.3%), and HIV/AIDS (0.2%). On the contrary, 58.2% of participants reported having more than one chronic disease.

### 3.3. Exposure Variables

The most used transportation to go to work was one’s own car (30.3%), followed by motorcycle (26.5%) and public transport (21.1%). The rest of the workers reported walking (10.2%), taxi (7.1%), bicycle (1.2%), or none (3.7%) to go to work. Almost half of the participants reported having contact with a person with suspected or confirmed COVID-19 infection. However, the presence of symptoms and hospitalization due to COVID-19 was low (Table 2); 91.7% reported using a face mask and only 9.5% used a face shield for protection.

### 3.4. Seroprevalence

**Figure 3:**
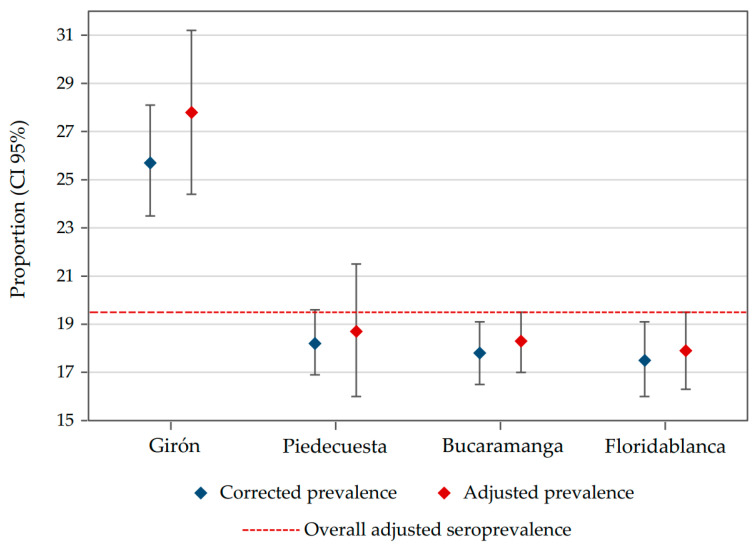
Seroprevalence by municipalities. Dashed line represents the overall adjusted seroprevalence estimated in our study. This line was added for comparison purposes.

**Figure 4:**
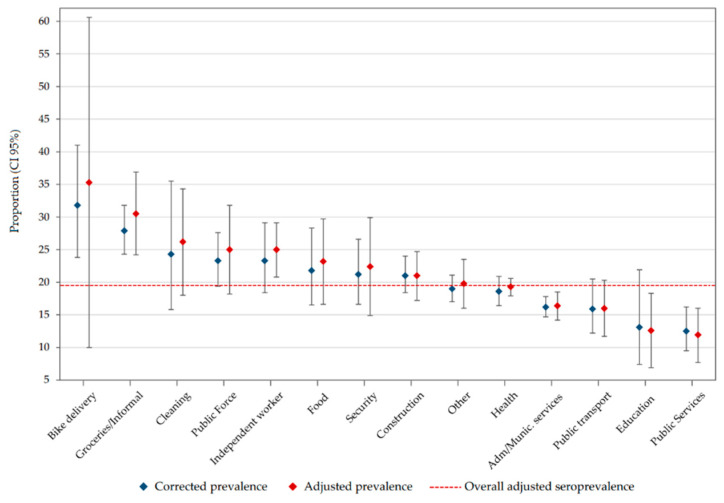
Seroprevalence according to occupational group. Dashed line represents the overall adjusted seroprevalence estimated in our study. This line was added for comparison purposes.

The overall corrected prevalence by study design was 18.8% (95% CI: 17.5–20.2), and adjusted for test performance was 19.5% (95% CI: 18.6–20.4). According to municipality, Girón had the greater adjusted seroprevalence (27.9%; 95% CI: 24.5–31.3), followed by Piedecuesta (18.8%; 95% CI: 16.0–21.5), Bucaramanga (18.3%; 95% CI: 17.0–19.6), and Floridablanca (17.9%; 95% CI: 16.4–19.5) (Figure 3 and Figure S2a,b). For occupational groups (Figure 4 and Table S1), those participants with multiple contact with other people during their working hours, such as motorcycle delivery workers, grocery store tenants, and informal commerce workers, had a higher frequency of seropositivity for SARS-CoV-2. While seroprevalence was similar among age groups (Supplemental Table S1), smoking status, and the presence of medical conditions (Table 2), it was higher in workers that used a bike (25.7%; 95% CI: 16.6–34.8), a motorcycle (24.0%; 95% CI: 22.1–25.9), and public transportation (23.9%; 95% CI: 21.8–26.0) than those using their own car (13.0%; 95% CI: 11.5–14.4) or a taxi (15.5%; 95% CI: 12.3–18.7) to go to work.

Participants that had symptoms related to COVID-19 since March 2020 presented an adjusted seroprevalence of 50.3% (95% CI: 47.9–52.7) vs. 57.7% (95% CI: 56.3–59.1) for those without symptoms, and participants that had contact with a person with suspected or confirmed COVID-19 infection presented an adjusted seroprevalence of 49.9% (95% CI: 48.1–51.7) vs. 41.8% (95% CI: 40.1–43.5) for those without contact.

**Figure 5:**
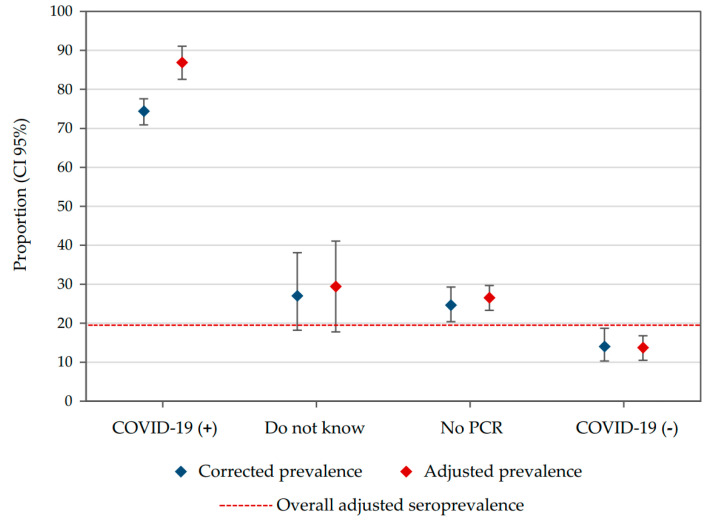
Seroprevalence according to self-reported COVID-19 previous diagnosis. Dashed line represents the overall adjusted seroprevalence estimated in our study. This line was added for comparison purposes.

On the contrary, for people with a previous confirmed diagnosis of COVID-19, the adjusted seroprevalence was 86.9% (95% CI: 82.6–91.1) compared to 13.7% (95% CI: 10.5–16.8) for those with a negative PCR (Figure 5). Given that our recruitment period was long, the effect of time on the probability of being seropositive was evaluated, but it was not statistically significant, even when adjusting by municipality, occupational group, age and sex (Supplemental Table S2).

## 4. Discussion

This study was the first to investigate seroprevalence in different occupational groups in the Bucaramanga metropolitan area (Santander, Colombia), under the concept of “super-spreaders” [23] to refer to individuals who, due to their work occupation, are in contact with many people during their working hours, which could facilitate the spread of the infection. Studying these workers is essential to improve the understanding of how the first peak of the pandemic occurred without being detected by the public health surveillance system. Our findings suggest that grocery store tenants, public forces (police and military personnel), informal commerce and independent workers, and delivery, food, cleaning, security, and construction workers played a special role in the SARS-CoV-2 transmission in the Bucaramanga metropolitan area. This could be a consequence of the informal sector not being able to fulfill the biosafety protocols required by the national government due to job and economic precariousness. Occupations with lower seroprevalence tend to be in the formal sector of the economy, indicating better working conditions and probably a higher socioeconomic status.

The results should be compared with caution, given that the transmission behavior of SARS-CoV-2 in Colombia was not homogeneous. The peaks that occurred were established at different times according to the severity of each department’s restrictions, especially mobility and closure of the different economic sectors. Until now, there have been few seroprevalence studies in Colombia. In the only published study with data from various regions of the country, 4740 workers participated, of whom only 23 were symptomatic. The data were collected between April and August 2020, and a seroprevalence close to 4% was observed [24]. According with non-published data, the “*Estudio País*” (“*Country Study*”), organized by the National Institute of Health, reported a high frequency, as in Leticia (59%; 95% CI: 54–65) and Barranquilla (55%; 95% CI: 51–61), while in other cities, the frequency was lower (Bogotá: 30%, 95% CI: 27–33; Medellín: 27%, 95% CI: 24–31; Bucaramanga: 32%, 95% CI: 29–36; unpublished data). Data collection was realized during the last trimester of 2020, and an in-house test was used for the analysis [25].

Local studies have been realized in Monteria and Bogotá. In Montería, 1368 individuals were randomly selected from the population, and between July and August 2020, a seroprevalence of 55.3% was estimated [26]; this was one of the highest seroprevalences in Colombia and Latin America for the time. A few small occupational studies have also been conducted. A study with 212 workers from the Bogotá international airport reported a seroprevalence of 16%, with samples collected between June and September 2020 [27]. In a university hospital in Bogotá, a study was carried out between June and August 2020 with 24 medical interns, 163 residents, and 164 medical doctors. The results indicated a seroprevalence of 8.3% [28]. A study with 237 students from a private university collected samples during the second semester of 2020 and found a seroprevalence of 13.5% [29].

Our estimate of general seroprevalence (19.8%) is lower than the estimates reported thus far for other studies conducted in Colombia and is very close to those reported for other countries such as Iran (22.16; 95% CI: 18.7–26.0) [30]. However, this difference could be related to the type of population included, given that in our study, we included subjects only over 18 years of age that reside and work (occupational sectors) in the previously mentioned municipalities, and that in *Estudio Pais*, Bucaramanga was the only city included, while in our study, the metropolitan area constituted by three other municipalities was evaluated.

Variations in seroprevalence for SARS-CoV-2 are very common, even in the same region or country. Rostami et al. [31] found a pooled seroprevalence for South America of 1.45 (95% CI: 0.95–1.94), including studies from Chile (10.78; 95% CI: 9.1–12.5) and Brazil (0.96; 95% CI: 0.52–1.40) conducted between March and May 2020 (0.222%; 95% CI: 0.107–0.408%) for Rio de Janeiro [12], where new daily cases were starting to rise. If we compare the estimate of general seroprevalence among the participating population in our study, it is higher than the estimates of seroprevalence in countries such as Spain (5.01; 95% CI: 4.83–5.18) or the United States (4.41%; 95% CI: 3.03–5.79). Therefore, it is more likely that the difference observed is related to the epidemic conditions of each territory and the restrictions approved in the sanitary regulations applied in each region than to the test’s characteristics [32].

Besides differences in location or type of population assessed in every study reported thus far, variations in seroprevalence could be also related to timing and the test used to assess immunoglobulins. In Bucaramanga, the first COVID-19 peak began at the end of July, with highest number of cases by mid-August, with a second wave after mid-November (~300 cases/day, when national estimates where conducted). However, no significant association has been reported between the incidence of COVID-19 cases and seroprevalence, such as reported by some authors [12]. On the contrary, the estimates of lower seroprevalence in our study compared to the National Seroprevalence Study could be related, in part, to the characteristics of the test used, given that it was an in-house test [33,34] and our study used a commercial test.

This study has its strengths. The sample size recruited is the largest one for a small non-capital city in Colombia and the estimation of seroprevalence was carried out in an adjusted way, considering complex design analysis and test performance. In addition, it is the only study that incorporated information from various occupations and that integrated epidemiological analysis with spatial analysis. However, this study has some limitations. Even though we sent out a wide invitation to several companies in the Bucaramanga metropolitan area, the most interested ones who agreed to participate in the survey were those providing health services. Other companies such as taxi drivers or bike messengers had low motivation to participate, despite the probability of high SARS-CoV-2 exposure. Additionally, our recruitment time frame was long for a seroprevalence study; however, and according to the National Health Institute, the daily report of new cases and deaths was constant in the four municipalities in this period. Additionally, we studied the effect of time (recruitment day) on the test results, and this was not statistically significant. An important point to note is that the tests used are not perfect and may lead to an underestimation of seroprevalence. This fact is well known, since even studies using the gold standard test (RT-qPCR) also underestimate the actual occurrence of infection [35].

## 5. Conclusions

The seroprevalence for SARS-CoV-2 in workers living in the Bucaramanga metropolitan area remains low, even below national estimates for this region. Moreover, given the variation in time and the type of population assessed, these results only reflect estimates for occupational groups in the four municipalities included. These results reinforce the variation in the frequency of seropositivity for the infection according to location, exposure, and moment of the epidemic curve, which makes it a challenge to find the true infection burden. Seroprevalence surveillance should be carried out periodically to better understand infection behavior and even to estimate seroconversion frequency and its related factors.

## Figures and Tables

**Table 1:** Sociodemographic variables of the study participants according to immunoglobulin type ^a^.

Variable	All	Negative	IgG+ Only	IgG/IgM+	IgM+ Only
*n*	7045	5738	677	434	196
Age (years, *n* = 6629) *	37.4	37.2	35.1	39.5	34.3
	(36.3–38.4)	(36.6–37.8)	(33.9–36.3)	(38.3–40.7)	(32.6–36.1)
Sex					
Men	2821 (40.0)	2308 (40.3)	264 (38.6)	186 (43.1)	63 (31.5)
Women	4219 (59.9)	3426 (59.6)	412 (61.2)	248 (56.9)	133 (68.5)
Other	5 (0.1)	4 (0.1)	1 (0.1)	-	-
Municipality					
Bucaramanga	3347 (47.5)	2752 (48.8)	310 (45.7)	202 (46.4)	83 (42.8)
Floridablanca	2226 (31.6)	1836 (25.5)	201 (23.0)	123 (21.9)	66 (26.4)
Girón	628 (8.9)	466 (11.9)	87 (18.5)	56 (18.6)	19 (14.1)
Piedecuesta	774 (10.9)	633 (13.8)	69 (12.5)	46 (13.0)	26 (16.5)
Other	70 (0.9)	-	-	-	-
Socioeconomic status					
1 (lowest)	575 (8.2)	412 (7.4)	94 (13.9)	48 (10.3)	21 (10.5)
2	1563 (22.2)	1175 (20.7)	213 (31.2)	126 (29.1)	49 (24.9)
3	2442 (34.6)	1977 (35.2)	240 (36.4)	154 (36.6)	71 (37.5)
4	1712 (24.3)	1496 (25.6)	102 (14.6)	78 (17.8)	36 (18.1)
5	395 (5.6)	352 (5.4)	15 (1.9)	17 (3.5)	11 (4.9)
6 (higher)	320 (4.5)	296 (4.9)	8 (1.1)	9 (1.9)	7 (3.3)
Unknown	38 (0.5)	30 (0.5)	5 (0.7)	2 (0.4)	1 (0.5)
Occupational sector					
Health	3295 (46.8)	2.697 (47.2)	309 (45.6)	183 (43.1)	106 (55.1)
Public transportation	282 (4.0)	237 (4.1)	25 (3.7)	11 (2.7)	9 (4.3)
Public forces (police/army)	148 (2.1)	114 (1.8)	17 (2.2)	15 (3.5)	2 (1.0)
Public services					
Security	242 (3.4)	213 (3.7)	19 (2.9)	8 (1.7)	2 (1.0)
Construction	114 (1.6)	91 (1.6)	10 (1.6)	6 (1.4)	7 (3.5)
Food	440 (6.2)	343 (6.0)	59 (8.2)	33 (6.9)	5 (2.2)
Education	151 (2.1)	118 (2.0)	18 (2.4)	13 (2.9)	2 (1.3)
Grocery store tenants/informal commerce	136 (1.9)	120 (2.0)	7 (1.2)	5 (1.0)	4 (2.2)
Independent worker	194 (2.7)	141 (2.4)	23 (3.4)	25 (5.7)	5 (2.5)
Administrative municipal services					
Cleaning	398 (5.6)	309 (5.1)	35 (5.2)	45 (10.5)	9 (3.8)
Bike delivery workers	1095 (15.5)	920 (15.9)	94 (13.8)	53 (12.2)	28 (14.1)
Other					
	106 (1.5)	79 (1.5)	15 (2.3)	9 (2.0)	3 (1.4)
	13 (0.2)	9 (0.1)	1 (0.1)	3 (0.6)	-
	422 (6.0)	338 (6.0)	45 (6.3)	25 (5.2)	14 (7.2)

^a^ Percentages in parentheses. * Mean (95% CI).

**Table 2:** Clinical and SARS-CoV-2 exposure variables according to immunoglobulin type ^a^.

Variable	All	Negative	IgG+ Only	IgG/IgM+	IgM+ Only
*n*	7045	5738	677	434	196
Smoking					
Yes (currently)	345 (4.9)	302 (5.3)	25 (3.8)	9 (1.9)	9 (4.3)
Yes (past)	1421 (20.2)	1.177 (20.3)	110 (15.9)	96 (22.1)	38 (19.2)
Yes (passive)	419 (5.9)	341 (6.0)	35 (5.1)	31 (7.1)	12 (6.6)
No	4860 (69.0)	3.918 (68.2)	507 (75.0)	298 (68.6)	137 (69.7)
Medical conditions					
Yes	1333 (18.9)	1.105 (18.9)	110 (15.7)	83 (18.6)	35 (17.3)
No	5509 (78.2)	4.474 (78.2)	549 (81.3)	332 (77.2)	154 (79.2)
Do not know	203 (2.9)	159 (2.8)	18 (2.8)	19 (4.2)	7 (3.3)
Contact with people with suspected or confirmed COVID-19					
Yes	3153 (44.8)	2.525 (43.9)	314 (46.4)	223 (52.0)	91 (45.0)
No	2898 (41.1)	2.411 (41.9)	262 (38.4)	144 (32.9)	81 (42.5)
Do not know	994 (14.1)	802 (14.0)	101 (15.1)	67 (15.0)	24 (12.4)
Symptoms related to COVID-19 since March 2020					
Yes	1643 (23.3)	1.074 (18.7)	297 (44.4)	230 (54.0)	42 (21.5)
No	5041 (71.5)	4.375 (76.1)	344 (50.3)	176 (39.7)	146 (74.4)
Do not know	361 (5.1)	289 (5.1)	36 (5.1)	28 (6.1)	8 (3.9)
Due to beginning of symptoms, COVID-19 diagnosis was confirmed					
Yes	401 (5.7)	101 (9.6)	159 (53.9)	130 (55.9)	11 (27.5)
No	474 (6.7)	405 (38.2)	25 (8.3)	25 (11.8)	19 (4.6)
No PCR	694 (9.8)	516 (48.5)	101 (34.6)	68 (30.1)	9 (22.1)
Do not know	56 (0.8)	40 (3.5)	8 (3.0)	6 (2.1)	2 (4.3)
Not applicable	5420 (76.9)				
Due to beginning of symptoms, was hospitalized for COVID-19 symptoms					
Yes	32 (0.4)	9 (0.6)	7 (2.4)	15 (5.7)	1 (1.3)
No	1846 (26.2)	1266 (99.1)	301 (97.1)	223 (93.5)	26 (98.6)
Do not know	4 (0.1)	2 (0.2)	-	2 (0.8)	-
Not applicable	5420 (76.9)				

^a^ Percentages in parentheses. COVID-19, coronavirus disease 2019.

## Data Availability

Data sharing not applicable.

## Supplementary Materials

The following are available online at https://www.mdpi.com/article/10.3390/ijerph18084172/s1, Figure S1: Geolocation process; Figure S2: Geolocation according to presence of antibodies and Masculinity index; Table S1: Estimates of corrected and adjusted seroprevalence; Table S2: Effect of days of recruitment on the test results.
