# Supplementary material for: Seroprevalence of SARS-CoV-2 Infection among Occupational Groups from the Bucaramanga Metropolitan Area, Colombia
Source: Int J Environ Res Public Health. 2021 Apr 15;18(8):4172. doi: 10.3390/ijerph18084172 (PMC8071134; doi:10.3390/ijerph18084172)
Supplement: Supplementary file 1 [file ijerph-18-04172-s001.zip › ijerph-1174066-supplementary.pdf]

Supplemental Materials

Seroprevalence of SARS-CoV-2 infection among occupational groups from Bucaramanga and metropolitan area, Colombia

Methods

Socioeconomic status definition

In Colombia, socioeconomic status ranged from 0 to 6, being 0 the most deprived and 6 the most affluent. This classification is made by national government according to 142 law from 1994. [1] Physical characteristics are the basis for assigning the strata to real estate residential areas of the country.

Strata 1, 2 and 3 correspond to low strata that house the users with fewer resources, who are beneficiaries of subsidies in the home public services; Strata 5 and 6 correspond to upper strata that house users with greater economic resources, who must pay extra costs (contribution) on the value of home public services. The Stratum 4 is not a beneficiary of subsidies, nor does it have to pay cost overruns, it pays exactly the value that the company defines as the cost of providing the service.

[1] https://www.dane.gov.co/index.php/servicios-al-ciudadano/servicios-informacion/estratificacion-socioeconomica#normatividad

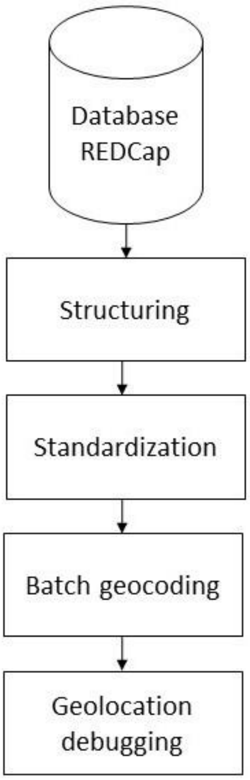

Figure S1. Geolocation process.

Table S1. Estimates of corrected and adjusted seroprevalence.

Variable	Corrected prevalence % (IC95%)	Adjusted prevalence % (IC95%)
Municipality		
Bucaramanga	17.8 (16.5 – 19.1)	18.3 (17.0 – 19.5)
Floridablanca	17.5 (16.0 – 19.1)	17.9 (16.3 – 19.5)
Girón	25.7 (23.5 – 28.1)	27.8 (24.4 – 31.2)
Piedecuesta	18.2 (16.9 – 19.6)	18.7 (16.0 – 21.5)
Age group		
18 a 29	20.0 (18.7 – 21.4)	20.1 (18.4 – 21.7)
30 a 39	19.0 (17.0 – 21.1)	19.2 (17.5 – 20.9)
40 a 49	17.9 (15.6 – 20.4)	17.2 (15.2 – 19.1)
50 a 59	17.2 (15.1 – 19.5)	17.0 (14.6 – 19.5)
60 a 69	17.0 (13.0 – 21.8)	16.1 (11.4 – 20.8)
70 and more	21.0 (14.7– 29.6)	21.5 (10.7 – 32.4)
Occupational sector		
Health,	18.6 (16.4 – 20.9)	19.3 (17.9 – 20.6)
Public transportation,	15.9 (12.2 – 20.5)	16.0 (11.7 – 20.3)
Public Force,	23.3 (19.4 – 27.6)	25.0 (18.2 – 31.8)
Public Services,	12.5 (9.5 – 16.2)	11.9 (7.7 – 16.0)
Security,	21.2 (16.6 – 26.6)	22.4 (14.9 – 29.9)
Construction,	21.0 (18.4 – 24.0)	21.0 (17.2 – 24.7)
Food,	21.8 (16.5 – 28.3)	23.2 (16.6 – 29.7)
Education,	13.1 (7.4 – 21.9)	12.6 (6.9 – 18.3)
Grocery store tenants/ Informal commerce,	27.9 (24.3 – 31.8)	30.5 (24.2 – 36.9)
Independent worker,	23.3 (18.4 – 29.1)	25.0 (20.8 – 29.1)
Adm/Municipal Services,	16.2 (14.7 – 17.8)	16.4 (14.2 – 18.5)
Cleaning,	24.3 (15.8 – 35.5)	26.2 (18.0 – 34.3)
Bike delivery workers,	31.8 (23.8 – 41.0)	35.3 (10.0 – 60.6)
Other	19.0 (17.0 – 21.1)	19.8 (16.0 – 23.5)

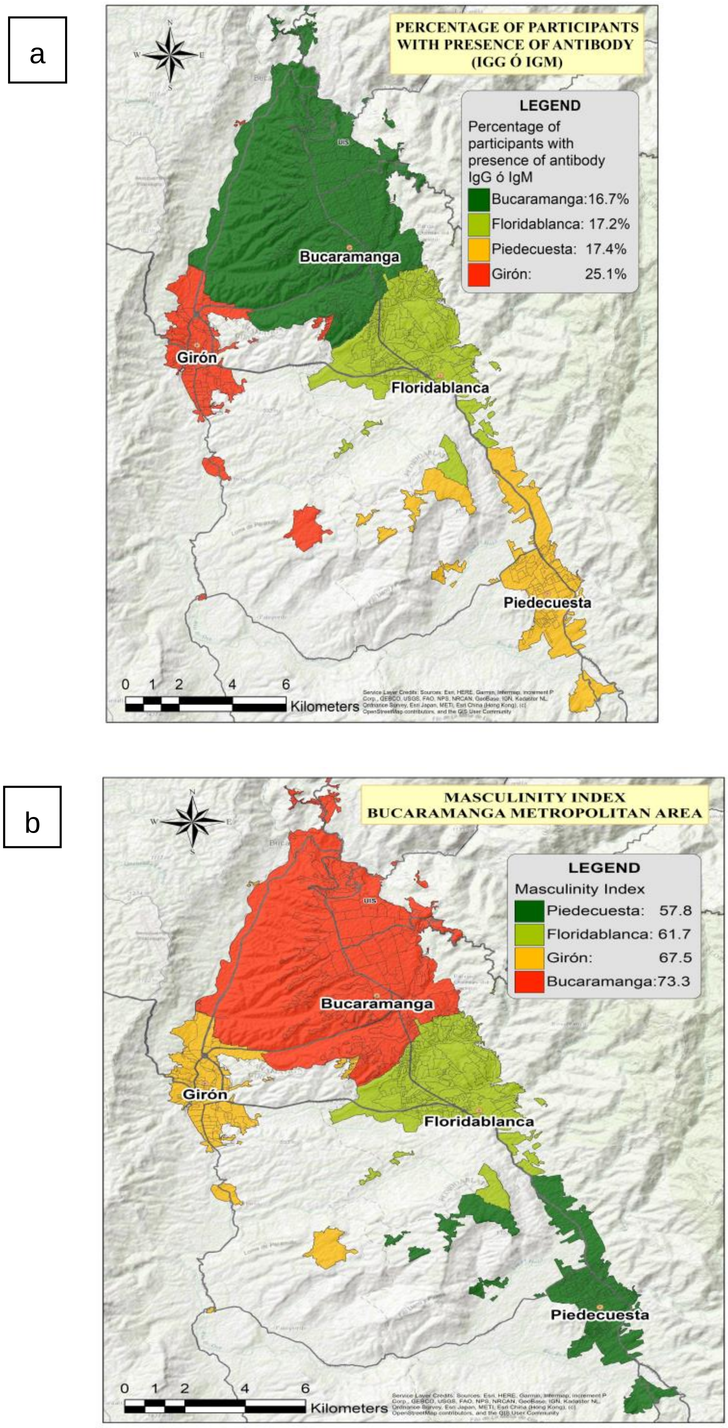

Figure S2. a. Percentage of participants with presence of antibody. b. Masculinity index.

Figure 2a represents the proportion of participants with positive IgG or IgM antibodies, the highest percentage of participants is found in the Girón, followed by Piedecuesta, Floridablanca, and Bucaramanga.

The masculinity index, commonly called sex ratio, is a demographic indicator that expresses the sex composition of a population. Figure 2b shows the ratio of men:women for each municipality, specifically the number of men participating for every 100 women, organized in cities from highest to lowest based on the indicator, it was found that Bucaramanga has the highest masculinity

index with 73.3 men per 100 women, followed by the municipality of Girón with 67.5, Floridablanca with 61.7, and Piedecuesta with 57.8.

Table S2. Effect of days of recruitment on test results.

**Model**	**Coef (IC95%)**
Test results + Days since recruitment began	0.0016 (-0.0011; 0.0043)
Test results + Days since recruitment began adjusted by municipality	0.0018 (-0.0006; 0.0042)
Test results + Days since recruitment began adjusted by municipality, occupational group	0.0016 (-0.0007; 0.0040)
Test results + Days since recruitment began adjusted by municipality, occupational group, age	0.0022 (-0.0005; 0.0050)
Test results + Days since recruitment began adjusted by municipality, occupational group, age, sex	0.0022 (-0.0005; 0.0050)
